# Supplementary material for: Tauroursodeoxycholic Acid Enhances Osteogenic Differentiation through EGFR/p-Akt/CREB1 Pathway in Mesenchymal Stem Cells
Source: Cells. 2023 May 24;12(11):1463. doi: 10.3390/cells12111463 (PMC10252885; doi:10.3390/cells12111463)
Supplement: Supplementary file 1 [file cells-12-01463-s001.zip › cells-2379288-supplementary.pdf]

Table S1. RT-PCR primer sequences

Gene	Primer sequence (5'-3') Forward	Reverse
ALP	CTCTTCGAGCCAGGGGACAT	GGTTCTTCCGCAGGATCTGG
COL1A1	AGGCTTCCCTGGTCTTCCTG	CCCCTCACGTCCAGATTCAC
BSP	ACAAGGCATAAACGGCACCA	CATTGTCTCCTCCGCTGCTG
OPN	CCTGAACGCGCCTTCTGATT	GCTTGTGGCTGTGGGTTTCA
OCN	AGGTGCAGCCTTTGTGTCCA	CAGGTAGCGCCTGGGTCTCT
OSX	TCTTCTGCGGCAAGAGGTTC	GCAGAGCAGGCAGGTGAACT
EGFR	TGCCGGAATGTCAGCCGAG	GGGTGGCACTGTATGCACTC
CREB1	ATTCGCACAGCACCCACTAGC	TGCTTCTTCAGCAGGCTGTGT
Cyclin D1	CCTCACACGCTTCCTCTCCA	GACTCCAGCAGGGCTTCGAT
Cyclin E1	TCAACGTGCAAGCCTCGGAT	ACTTCCTCTCTATTTGCCCAGCT
GAPDH	TTGCCCTCAACGACCACTTT	CATGAGGTCCACCACCCTGT
